# Miller-Fisher Syndrome: Is the ataxia central or peripheral?

**DOI:** 10.1186/s40673-015-0021-3

**Published:** 2015-03-01

**Authors:** Robert D Sandler, Nigel Hoggard, Marios Hadjivassiliou

**Affiliations:** Academic Department of Neurosciences, Royal Hallamshire Hospital, Glossop Road, Sheffield, S10 2JF UK

**Keywords:** Miller-Fisher, Ataxia, Magnetic resonance spectroscopy, Cerebellum

## Abstract

A 50-year-old man presented with a brief history of slurred speech, unsteadiness, double vision and paraesthesia. He had been unwell for 12 days with campylobacter gastroenteritis. On examination, there was ophthalmoplegia, nystagmus, areflexia and lower limb and gait ataxia. Serological testing was positive for GQ1b antibody in keeping with the diagnosis of Miller Fisher Syndrome (MFS). He was treated with two courses of intravenous immunoglobulins and made a good recovery, only displaying mild gait ataxia when reviewed in clinic 2.5 months later.

There has long been a debate as to whether the ataxia in MFS originates in the cerebellum or it is more peripheral. In this case, magnetic resonance spectroscopy (MRS) revealed a reduced NAA/Cr ratio in the cerebellar vermis and right cerebral hemisphere, suggestive of cerebellar dysfunction. The NAA/Cr normalised 2.5 months later reflecting the clinical recovery. The findings on MRS suggest that the cerebellum is involved in MFS.

## Background

Miller Fisher syndrome (MFS) is a variant of Guillain-Barre Syndrome (GBS), accounting for approximately 5% of acute inflammatory polyneuropathies. The predominant features of MFS are ophthalmoplegia and ataxia, with a peripheral neuropathy being only a very mild clinical feature. This is in contrast to GBS, where weakness and sensory disturbance are usually the presenting features [1,2]. Ever since the first publications of cases of MFS there has been a debate as to whether the ataxia is central or peripheral in origin. We report a patient with some evidence in support of cerebellar involvement.

## Case report

### Clinical case

A 50-year-old manual laborer presented to the Emergency Department with a 24-hour history of slurred speech, unsteadiness, double vision and pin & needles affecting both hands. He was also complaining of nasal regurgitation of fluids. The only past medical history was of stage 1 hypertension, for which he was on no medication. He had been feeling generally unwell for the past 12 days and had non-bloody diarrhea. Stool culture performed by his General Practitioner grew Campylobacter jejuni. He was not treated with antibiotics.

On admission, he was noted to have a nasal speech. There was poor palatal elevation. He had limitation of eye movement and nystagmus on left lateral gaze. The patient reported no diplopia during the examination. There was no peripheral motor deficit. Knee and triceps reflexes were absent bilaterally with wrist, bicep and ankle reflexes being present. Plantars were down going. There was some patchy sensory loss. He had dysdiadochokinesia, lower limb and gait ataxia.

Cerebrospinal fluid (CSF) analysis showed mildly elevated protein (0.5 mg/L, upper limit of normal 0.45 mg/L). Serological testing showed positive GQ1B antibodies in support of the diagnosis of MFS. Results of nerve conduction studies were consistent with a diagnosis of MFS showing mild disturbance in sensory conduction parameters with normal motor responses. There was no evidence of demyelination. Speech and Language Therapy assessment recommended nasogastric feeding due to the unsafe swallow and eventually the patient required a percutaneous endogastric tube for feeding.

Magnetic resonance spectroscopy (MRS) of the brain was performed, which identified reduced N-acetylaspartate/creatine (NAA/Cr) ratio of 0.9 at the superior cerebellar vermis, and 0.86 at the right cerebellar hemisphere (in healthy individuals both values should be above 1).

He was treated with a 5-day course of Intravenous Immunoglobulins (IVIg). His FVC was 3.13 L during treatment and he did not report any respiratory problems. Due to slow recovery and feeding difficulties he had a further 5-day course of IVIg 2 weeks later. His FVC and mobility improved. His speech returned to normal and he was transferred to a local rehabilitation unit. On discharge, he still complained of blurred vision, which was attributed to incompletely resolved ophthalmoplegia.

He was reviewed in clinic after discharge from the rehabilitation centre 2.5 months after the acute admission. Clinical examination showed normal eye movements with no residual limb ataxia. He still had some problems with mild gait ataxia as evident by mild difficulty in tandem walking. Repeat MRS at that stage showed normalization of NAA/Cr from the hemisphere at 1.01 and a similar value from the vermis perhaps reflecting the residual gait ataxia. A further review of the patient 3 months later showed complete resolution of the gait ataxia.

### Discussion

There has been much debate as to whether the ataxia in patients with MFS arises from cerebellar pathology (central) or from dysfunction of cerebellar afferents with involvement of the proprioceptive spinocerebellar pathways (peripheral). Existing case reports have commented on abnormalities found in the medulla and pons, indicating a central nervous system pathology and also evidence of peripheral neuropathy on neurophysiology, however there are no reports of cerebellar abnormalities. The majority of case reports describe normal appearances on magnetic resonance imaging, although no MRS was performed [3-8]. Some cases do report central involvement, however this is not specifically cerebellar [9,10].

From the original work of Fisher, and from subsequent publications, it was suggested that the cause of ataxia might be due to selective involvement of Ia afferent neurons along their path from muscle spindles to the spinal cord [1,11]. The GQ1b antigen is highly expressed in the oculomotor, trochlear and abducens nerves as well as in muscle spindles in the limbs [12].

Evidence of direct cerebellar involvement has also been proposed. For example Kim *et al.* have demonstrated cerebellar (vermis and hemispheres) glucose hypermetabolism in 10 patients with MFS [13]. This normalized after clinical recovery. Immunohistochemistry of human cerebellum using sera from patients with MFS showed selective staining of the cerebellar molecular layer [14]. Pathological findings are limited due to the good prognosis associated with MFS. However evidence of loss of purkinje neurons in the cerebellum in patients with MFS has been reported in autopsy specimens [15].

This report supports cerebellar involvement in MFS in addition to the peripheral neuropathy. In MR spectroscopy, neuronal levels of creatine are stable, serving as a reference point to measure the concentration of NAA, which is decreased in malfunctioning neurons [12].Therefore, the NAA/Cr ratio, obtained from MRS with the voxel placed over the vermis and the cerebellar hemispheres is a useful tool in identifying neuronal dysfunction and monitoring progression or improvement of neuronal function [16-20]. In this case, MRS identified significant reduction of NAA/Cr in both the vermis and the right hemisphere whilst the patient was symptomatic with normalization of the NAA/Cr 2.5 months later. The change in MRS was significant [21].

## Conclusion

Miller Fisher syndrome is associated with direct cerebellar involvement in addition to the peripheral neuropathy. This was demonstrated by abnormal spectroscopy of the cerebellum during clinical evidence of ataxia and normalization of the spectroscopy on clinical recovery.

## Consent

Written informed consent was obtained from the patient for publication of this Case report and any accompanying images. A copy of the written consent is available for review by the Editor-in-Chief of this journal.

## Abbreviations

MFS: Miller-Fisher Syndrome
GBS: Guillian-Barre Syndrome
FVC: Forced vital capacity
CSF: Cerebrospinal fluid
MRS: Magentic resonance spectroscopy
NAA:Cr: N-acetylaspartate to creatine ratio
IVIg: Intravenous immunoglobulins
